# Cysteinyl Maresins Reprogram Macrophages to Protect Mice from Streptococcus pneumoniae after Influenza A Virus Infection

**DOI:** 10.1128/mbio.01267-22

**Published:** 2022-08-01

**Authors:** Luciana P. Tavares, Thayse R. Brüggemann, Rafael M. Rezende, Marina G. Machado, R. Elaine Cagnina, Ashley E. Shay, Cristiana C. Garcia, Julie Nijmeh, Mauro M. Teixeira, Bruce D. Levy

**Affiliations:** a Pulmonary and Critical Care Medicine Division, Department of Medicine, Brigham and Women’s Hospital, Harvard Medical School, Boston, Massachusetts, USA; b Laboratório de Imunofarmacologia, Departamento de Bioquímica e Imunologia, Instituto de Ciências Biológicas, Universidade Federal de Minas Gerais, Belo Horizonte, Minas Gerais, Brazil; c Ann Romney Center for Neurologic Diseases, Department of Neurology, Brigham and Women’s Hospital, Harvard Medical School, Boston, Massachusetts, USA; d Center for Experimental Therapeutics and Reperfusion Injury, Department of Anesthesiology, Perioperative and Pain Medicine, Brigham and Women’s Hospital, Harvard Medical School, Boston, Massachusetts, USA; e Laboratório de Vírus Respiratórios e do Sarampo, Instituto Oswaldo Cruz (Fiocruz), Rio de Janeiro, Rio de Janeiro, Brazil; University of Pittsburgh School of Medicine

**Keywords:** influenza A virus, *Streptococcus pneumoniae*, resolution of inflammation, proresolving mediators, maresin conjugates in tissue regeneration (MCTRs), alveolar macrophages, secondary infection

## Abstract

Influenza A virus (IAV) infections are a leading cause of mortality worldwide. Excess mortality during IAV epidemics and pandemics is attributable to secondary bacterial infections, particularly pneumonia caused by Streptococcus pneumoniae. Resident alveolar macrophages (rAMs) are early responders to respiratory infections that coordinate initial host defense responses. Maresin conjugates in tissue regeneration (MCTRs) are recently elucidated cysteinyl maresins that are produced by and act on macrophages. Roles for MCTRs in responses to respiratory infections remain to be determined. Here, IAV infection led to transient decreases in rAM numbers. Repopulated lung macrophages displayed transcriptional alterations 21 days post-IAV with prolonged susceptibility to secondary pneumococcal infection. Administration of a mix of MCTR1 to 3 or MCTR3 alone post-IAV decreased lung inflammation and bacterial load 48 and 72 h after secondary pneumococcal infection. MCTR-exposed rAMs had increased migration and phagocytosis of Streptococcus pneumoniae, reduced secretion of CXCL1, and a reversion toward baseline levels of several IAV-induced pneumonia susceptibility genes. Together, MCTRs counter regulated post-IAV changes in rAMs to promote a rapid return of bacteria host defense.

## INTRODUCTION

Influenza A virus (IAV) infection leads to substantial mortality and morbidity worldwide, accounting for up to 5 million severe cases annually (1). While most cases of IAV infection do not cause death, secondary bacterial infections are a major factor contributing to worse prognoses and increased hospitalization (2, 3). Streptococcus pneumoniae is one of the leading causes of post-IAV secondary pneumonia and, during IAV pandemics, pneumococcal pneumonia is responsible for a large proportion of IAV-associated deaths (2). Despite the availability of antibiotics and vaccines, incidences of post-IAV pneumonia and critical illness remain high (4). Excess risk for secondary bacterial pneumonia after severe respiratory viral infection is also observed during SARS-CoV-2 infections (5); therefore, host-directed approaches to enhance lung bacterial defense mechanisms are of broad importance.

After IAV infection, several host responses to the virus have been identified that increase susceptibility to pneumococcus pneumonia, most notably dysregulation of innate immune responses that are engaged to control IAV infection (6). Resident alveolar macrophages (rAMs) are early airway responders to pathogen invasion of the lower respiratory tract (7). IAV infection can impact rAM viability and/or function (8, 9), and depletion of murine rAMs during IAV infection is associated with increased susceptibility to pneumococcal pneumonia (8). In addition to controlling bacterial proliferation, rAMs are important for regulating inflammatory responses in the lung during infections (10).

Resolution of lung inflammation is an active process that regulates pathogen elimination, host defense, and clearance of inflammatory cells and debris from infected tissues to restore organ homeostasis (11). Specialized proresolving mediators (SPMs) are a class of bioactive autacoids that terminate inflammatory responses and signal for resolution (11). Recently, a new family of cysteinyl-containing (cys)-SPMs that are produced by mouse and human macrophages was discovered and named the maresin conjugates in tissue regeneration (MCTRs), e.g., cys-Maresins (12). MCTR1 (13*R*-glutathionyl,14*S*-hydroxy-4*Z*,7*Z*,9*E*,11*E*,16*Z*,19*Z*-docosahexaenoic acid) is produced from docosahexaenoic acid (DHA) via the biosynthetic enzymes 12-lipoxygenase (Alox12) and glutathione *S*-transferase Mu 4 (GSTM4) (13). MCTR1 is subsequently enzymatically converted to MCTR2 (13*R*-cysteinylglycinyl,14*S*-hydroxy-4*Z*,7*Z*,9*E*,11*E*,16*Z*,19*Z*-docosahexaenoic acid) by gamma-glutamyl transferase and then MCTR3 (13*R*-cysteinyl,14*S*-hydroxy-4*Z*,7*Z*,9*E*,11*E*,13*R*,14*S*,16*Z*,19*Z*-docosahexaenoic acid) by dipeptidases in mouse and human lungs (14). Of note, levels of Alox12-derived lipid mediators are inversely correlated with IAV severity in humans (15). All three MCTRs mediate proresolving and tissue reparative bioactions, including macrophage phagocytosis and efferocytosis (13, 16) and display protective actions in allergic lung inflammation and airway contraction (14). Thus, macrophages can produce and respond to MCTRs based on their autacoid properties (13, 16). Here, the prolonged post-IAV increase in susceptibility to pneumococcal infection was associated with changes in rAM gene expression and cellular dysfunction. Administration post-IAV of a mix of MCTR1, MCTR2, and MCTR3 or MCTR3 alone significantly enhanced pneumococcal host defense and decreased pathogen-initiated lung inflammation, in part by reversing the IAV-induced changes in rAM phenotype and function.

## RESULTS

### Influenza A virus infection leads to prolonged increased host susceptibility to pneumococcal infection.

**FIG 1:**
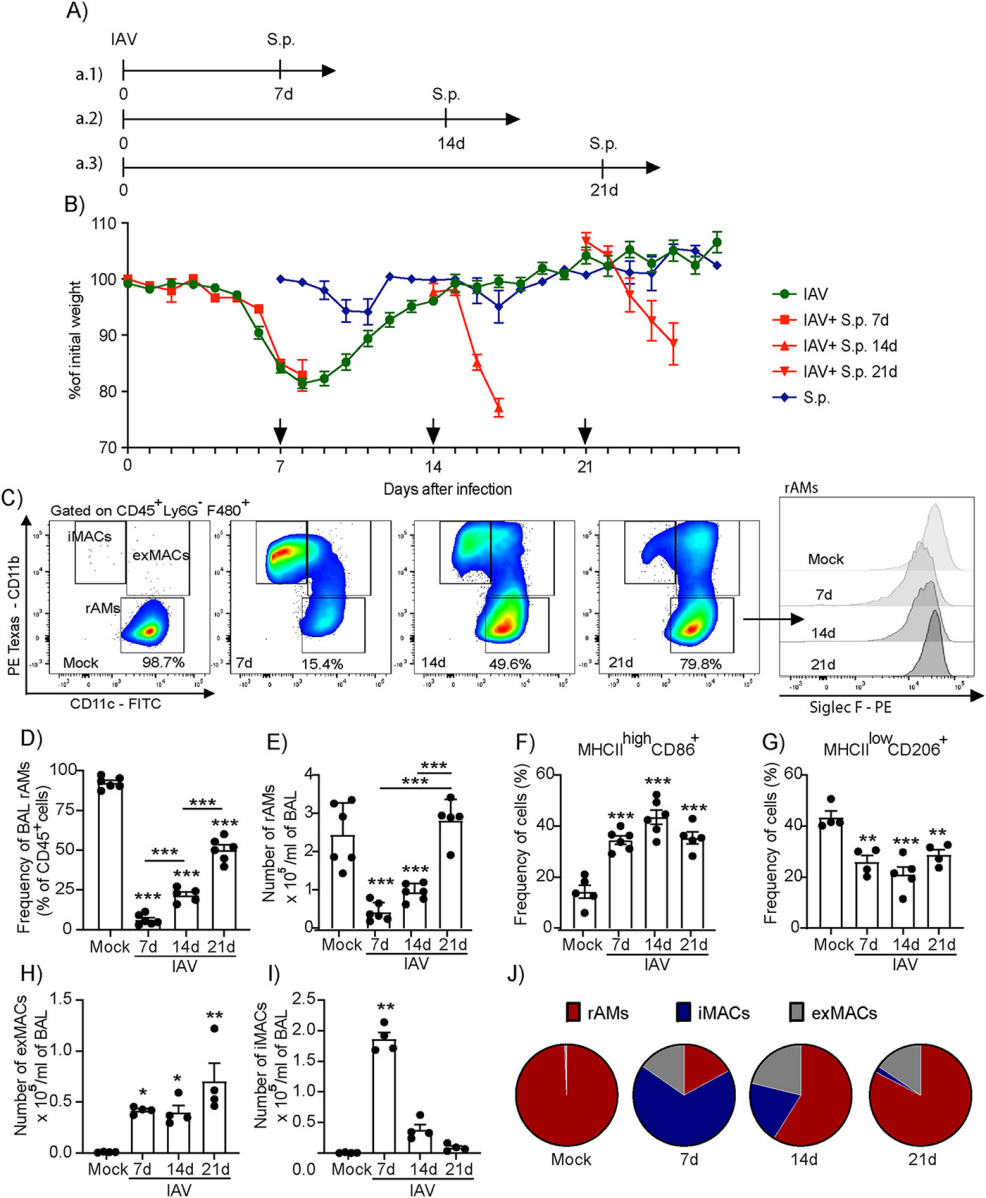
Influenza A virus infection leads to prolonged increased host susceptibility to pneumococcal infection. (A) C57BL/6 mice were infected with a nonlethal inoculum of IAV (500 PFU, i.n.). On days 7 (a.1), 14 (a.2), or 21 (a.3) days after IAV infection, three groups of animals were secondarily infected with S. pneumoniae (serotype 3; 10^3^ CFU, i.n.). (B) Animals were weighed daily until day 28 or death (arrows indicate the timing of S. pneumoniae infection). (C to I) On days 7, 14, and 21 after IAV infection, BAL macrophages were characterized and quantified by flow cytometry as resident alveolar macrophages (rAMs) (CD45^+^ Ly6G^−^ F4/80^+^ CD11b^−^ CD11c^+^ SiglecF^+^), exudative macrophages (exMACs) (CD45^+^ Ly6G^−^ F4/80^+^ CD11b^+^ CD11c^+^), inflammatory macrophages (iMACs) (CD45^+^ Ly6G^−^ F4/80^+^ CD11b^+^ CD11c^−^), and frequency of M1 (MHCII^high^ CD86^+^) and M2-like (MHCII^low^ CD206^+^) cells among rAMs. Uninfected mice were used as control (mock). Results are expressed as mean ± SEM; *n* = 5 mice in two independent experiments. *, *P* < 0.05; **, *P* < 0.01; ***, *P* < 0.001 by one-way ANOVA. (J) The proportion of total lung macrophages at baseline and days 7, 14, and 21 post-IAV infection. *n* = 4 mice, representative of two independent experiments, with rAMs in red, iMACs in blue, and exMACs in gray.

To investigate the impact of IAV lung infection on host susceptibility to secondary pneumococcal pneumonia, we infected mice with a nonlethal inoculum of IAV (A/WSN/33 H1N1, 500 PFU, intranasally [i.n.]), followed by S. pneumoniae (10^3^ CFU, i.n.) at 7, 14, or 21 days post-IAV (Fig. 1A). Following infections with IAV alone, the weight of infected animals began decreasing 5 days post-IAV infection, reached a nadir at day 8, and recovered fully to baseline 14 days post-IAV (Fig. 1B). These changes in weight qualitatively mirrored the changes in animal activity, as the animals no longer appeared ill 14 days post-IAV. Of note, IAV infection was cleared at day 14 in this model (see Fig. S1A in the supplemental material).

10.1128/mbio.01267-22.1 FIG S1 Kinetics of IAV *M1* expression and gating strategy for alveolar macrophages. (A) qPCR for IAV *M1* was performed in lungs from IAV-infected (500 PFU) or uninfected (mock) mice. (B) Representative flow cytometry pseudocolor plots of BAL cells obtained from uninfected mice (mock) and IAV-infected mice on days 7, 14, and 21 post-IAV infection. Download FIG S1, TIF file, 1.6 MB. Copyright © 2022 Tavares et al. 2022 Tavares et al. https://creativecommons.org/licenses/by/4.0/ This content is distributed under the terms of the Creative Commons Attribution 4.0 International license.

To ascertain susceptibility to bacterial pneumonia during active IAV infection, after viral clearance and then an additional week later after apparent resolution, mice were exposed to S. pneumoniae (10^3^ CFU, i.n.) at 7, 14, or 21 days post-IAV infection, respectively (Fig. 1A). This inoculum of S. pneumoniae without prior IAV infection did not cause weight loss and was generally well tolerated by mice (Fig. 1B, blue line). When given at the peak of illness, 7 days post-IAV, this S. pneumoniae inoculum led to significant weight loss and 100% mortality within 48 h (Fig. 1B). When given after viral clearance (Fig. S1A), when mice had recovered to their baseline weight and behavior, the same inoculum of S. pneumoniae at 14 days post-IAV led to progressive weight loss and mice reached criteria for euthanasia within 4 days after bacterial infection (Fig. 1B). Of interest, even 21 days post-IAV infection, mice were severely affected by this modest inoculum of S. pneumoniae with progressive weight loss and decreased activity that met criteria for euthanasia within 5 days (Fig. 1B). These results demonstrate a marked and prolonged alteration in host susceptibility to S. pneumoniae lung infection after IAV (A/WSN/33 H1N1) infection.

### Resident alveolar macrophages are significantly decreased during IAV infection and repopulated over time by macrophages with a distinct activated phenotype.

Alveolar macrophages are host defense sentinels for S. pneumoniae (17). To investigate the impact of IAV infection on rAMs, cells were collected by bronchoalveolar lavage (BAL) at days 0, 7, 14, and 21 post-IAV infection and were assessed by flow cytometry (Fig. 1C). rAM (CD45^+^ F4/80^+^ CD11b^−^ CD11c^+^ SiglecF^+^) frequency was significantly reduced 7 days post-IAV infection (Fig. 1D), and rAM numbers were markedly decreased from 2.45 (±0.83) × 10^5^ cells at day 0 to 0.43 (±0.24) × 10^5^ cells at day 7 (Fig. 1E). rAM numbers increased from day 7 to day 14 (0.94 [±0.22] × 10^5^ cells) and increased even further by day 21, returning to approximately baseline numbers (2.82 [±0.83] × 10^5^ cells) (Fig. 1E; see also Fig. S1B). While rAM numbers had normalized by day 21 after IAV infection, the cells were still polarized, continuing to express high levels of major histocompatibility complex class II (MHCII) and CD86, typical markers of proinflammatory M1 macrophages (18) (Fig. 1F). In contrast, the frequency of rAMs that were MHCII^low^ and CD206^+^, markers of counterregulatory M2 macrophages (19), remained decreased 21 days after infection (Fig. 1G). In addition to rAMs, lung infection leads to the recruitment of additional macrophage subsets, including exudative and infiltrating macrophages (exMACs and iMACs, respectively) (20). While the numbers of rAMs were decreased post-IAV infection, the numbers of exMACs were increased at days 7, 14, and 21 post-IAV infection (Fig. 1H), and the numbers of iMACs were increased at day 7 post-IAV infection, decreasing thereafter at days 14 and 21 to baseline levels (Fig. 1I). Of note, 21 days post-IAV infection or 48 h after S. pneumoniae infection, singly infected mice did not show significant increases in BAL neutrophils or bacteremia (see Fig. S2 in the supplemental material), suggesting that these macrophages or other lung resident cells play important roles in the susceptibility observed in the doubly infected animals. Together, these data indicate that different populations of alveolar macrophages are present in the lungs post-IAV infection (Fig. 1J) and that the rAMs and monocyte-derived macrophages in the airways during the first 3 weeks after IAV infection have an activated phenotype.

10.1128/mbio.01267-22.2 FIG S2 Immunophenotyping of mice after single infection with IAV or S. pneumoniae. C57BL/6 mice were infected with IAV (500 PFU), and BAL was performed at day 21 for immunophenotyping by flow cytometry. Separately, mice were infected with S. pneumoniae (1,000 CFU) and immunophenotyped on day 2 postinfection. Uninfected animals were used as controls (mock). BAL total leukocytes (A), neutrophils (B), rAMs (C), iMACs (D), and exMACs (E) were enumerated after infection (IAV, day 21; S. pneumoniae, day 2). (F) BALF total protein was determined by Bradford assay. (G) BAL fluid, lung, and blood CFUs were determined on day 2 post-S. pneumoniae infection (see Materials and Methods). Results are expressed as mean ± SEM; *n* = 5 mice. *, *P* < 0.05; **, *P* < 0.01 by One-way ANOVA test. Download FIG S2, TIF file, 1.1 MB. Copyright © 2022 Tavares et al. 2022 Tavares et al. https://creativecommons.org/licenses/by/4.0/ This content is distributed under the terms of the Creative Commons Attribution 4.0 International license.

### IAV infection induces prolonged transcriptional changes in rAMs.

**FIG 2:**
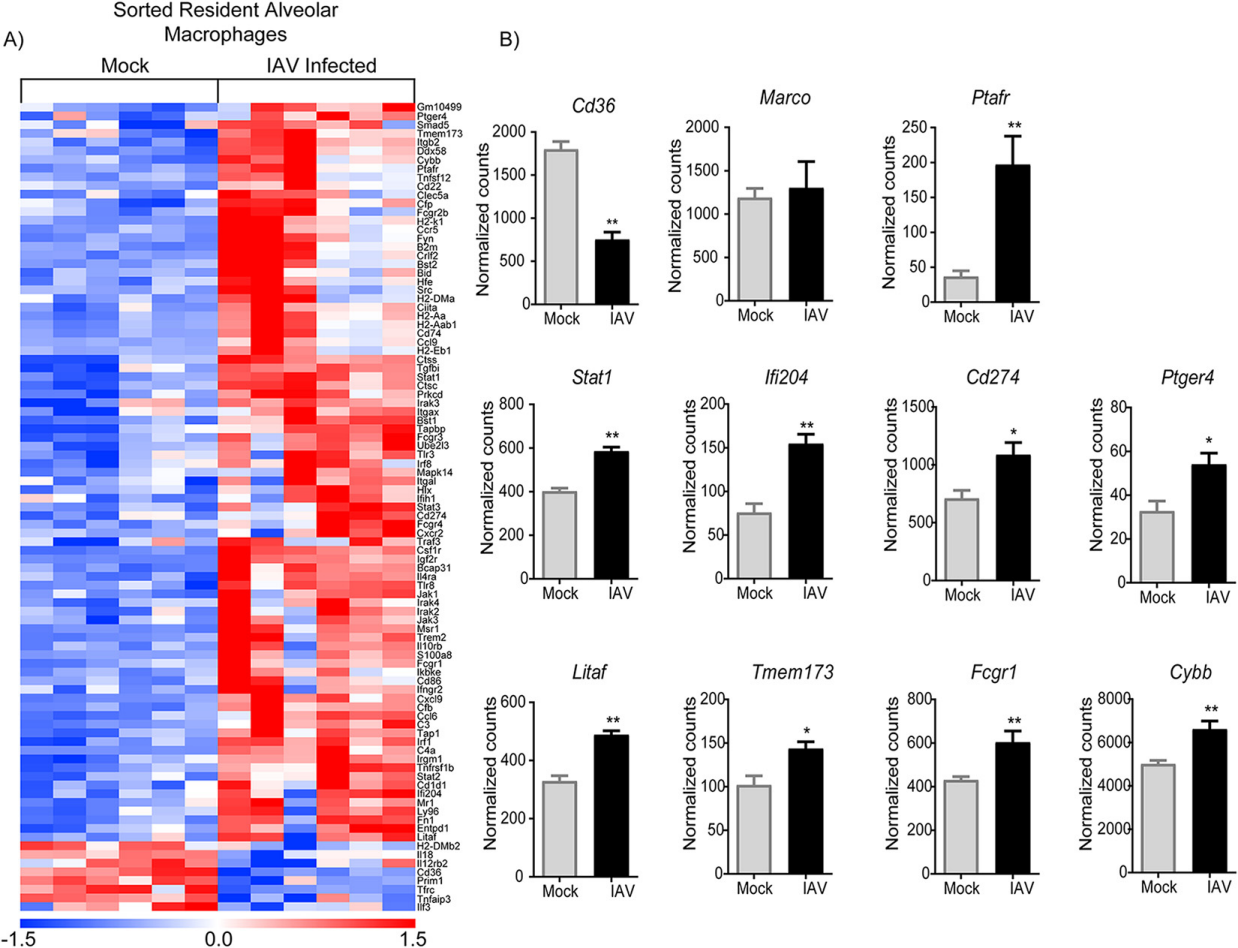
IAV infection induces prolonged transcriptional changes in rAMs. (A) Heatmap showing gene expression correlation between sorted rAMs (CD45^+^ F4/80^+^ CD11b^−^ CD11c^+^ SiglecF^+^) from uninfected (mock) and IAV-infected (21 days post-IAV infection) mice. (B) Representative genes that were differentially expressed after IAV infection. Results are presented as the normalized mRNA counts obtained. Each column in the heatmap is the result of rAMs obtained and pooled from 4 mice. Color saturation represents the magnitude of gene expression change, with full intensity at 1.5-fold up- or downregulation. Data are presented as mean ± SEM for *n* = 6 pooled samples of rAMs, *, *P* < 0.05; **, *P* < 0.01 by unpaired Mann-Whitney test.

To characterize the persistent activated rAM immunophenotype more comprehensively 21 days post-IAV infection, rAMs were sorted by fluorescence-activated cell sorter (FACS) and mRNA profiled using the NanoString Immunology codeset. The rAMs collected by BAL from infected animals 21 days post-IAV clustered in a clearly distinct group from control uninfected rAMs (Fig. 2A). Of note, among the genes assessed by NanoString, 8 genes were significantly downregulated, while 86 genes were significantly upregulated 21 days post-IAV (*P* < 0.05). Based on prior reports of host susceptibility factors for bacterial infection (21–24), rAM expression of several potentially relevant genes was altered post-IAV infection, including pathogen-related receptors (i.e., *Cd36* and *Ptafr*), interferon induced genes (i.e., *Stat1* and *If204*), and inflammatory response-related genes (i.e., *Cd274*, *Ptger4*, *Litaf*, *Tmem173*, *Fcgr1*, and *Cybb*) (Fig. 2B). Macrophage phagocytosis of S. pneumoniae proceeds in part via the scavenger receptor CD36 (23), and rAM expression of CD36 was significantly downregulated 21 days post-IAV infection (Fig. 2). In addition, genes related to antiviral and inflammatory responses were upregulated in rAMs 21 days post-IAV infection (Fig. 2B). Together, these data provide evidence for potential mechanisms for susceptibility to S. pneumoniae infection via their rAM-activated phenotype in the lungs 21 days post-IAV and more generally suggest that these activated macrophages had yet to assume a proresolving state.

### MCTRs are protective from secondary pneumococcal pneumonia post-IAV infection.

**FIG 3:**
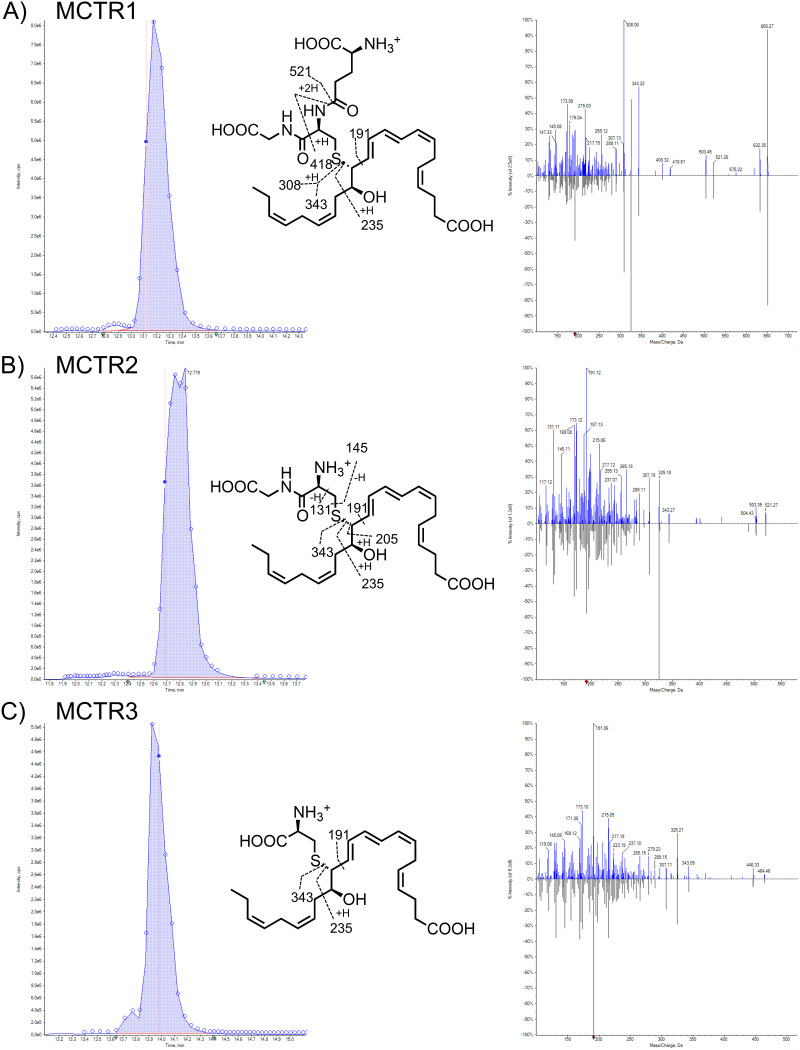
Structural authentication of MCTR1, MCTR2, and MCTR3. MCTRs were each confirmed by matching their retention times to those of reference materials and unbiased library fit scores. Screen captures from Sciex OS-Q version 1.7.0.36606 of targeted MRM (left) and EPI (right) scans of MCTR1 (*m/z* 650 > 191) (inset, MCTR1 structure with proposed fragmentation and a 98.9% unbiased library fit score) (A), MCTR2 (*m/z* 521 > 191) (inset, MCTR2 structure with proposed fragmentation and a 96.9% unbiased library fit score) (B), and MCTR3 (*m/z* 464 > 191) (inset, MCTR3 structure with proposed fragmentation and a 99.0% unbiased library fit score) (C). For MRM scan screen captures, the arrows along the *x* axis indicate the retention times at which the quantification (shaded) began and ended. The filled datapoint indicates the intensity and retention time (line) the EPI scan (right) was captured. For EPI scan screen captures, the arrow along the *x* axis indicates Q3. The default values of 0.00^th^ for *m/z* are shown. However, the accuracy of this MS/MS instrument is estimated at approximately *m/z* ±0.01. The upper spectrum is from the material for validation, and the lower spectrum is from the custom cys-SPM spectral MS/MS library.

Recently, the cys-SPM family of MCTRs were shown to enhance macrophage phagocytosis of bacteria (16) and promote the resolution of sterile lung inflammation (14). To determine whether MCTRs could protect IAV-infected mice from secondary pneumococcal pneumonia, we next administered exogenous MCTRs post-IAV infection and prior to S. pneumoniae exposure. MCTR1, MCTR2, and MCTR3 were prepared by total organic synthesis (13), characterized, and validated, employing liquid chromatography with tandem mass spectrometry (LC-MS/MS). MCTR1, MCTR2 and MCTR3 each gave matched retention times and unbiased fit scores of >96% to synthetic reference materials (Fig. 3). The enhanced product ion (EPI) scan of MCTR1 gave a parent ion of *m/z* 650 = M+H and daughter ions at *m/z* 632 = M+H-H_2_O, *m/z* 521, *m/z* 503 = 521-H_2_O, *m/z* 418, *m/z* 343, *m/z* 308, *m/z* 217 = 235-H_2_O, *m/z* 191, *m/z* 173 = 191-H_2_O, and *m/z* 147 = 191-CO_2_ (Fig. 3A). The EPI scan of MCTR2 gave a parent ion of *m/z* 521 = M+H and daughter ions of *m/z* 503 = M+H-H_2_O, *m/z* 343, *m/z* 325 = 343-H_2_O, *m/z* 217 = 235-H_2_O, *m/z* 191, *m/z* 187 = 205-H_2_O, *m/z* 173 = 191-H_2_O, *m/z* 145, and *m/z* 131 (Fig. 3B). The EPI scan of MCTR3 gave a parent ion of *m/z* 464 = M+H and daughter ions of *m/z* 446 = M+H-H_2_O, *m/z* 343, *m/z* 325 = 343-H_2_O, *m/z* 217 = 235-H_2_O, *m/z* 191, and *m/z* 173 = 191-H_2_O (Fig. 3C).

**FIG 4:**
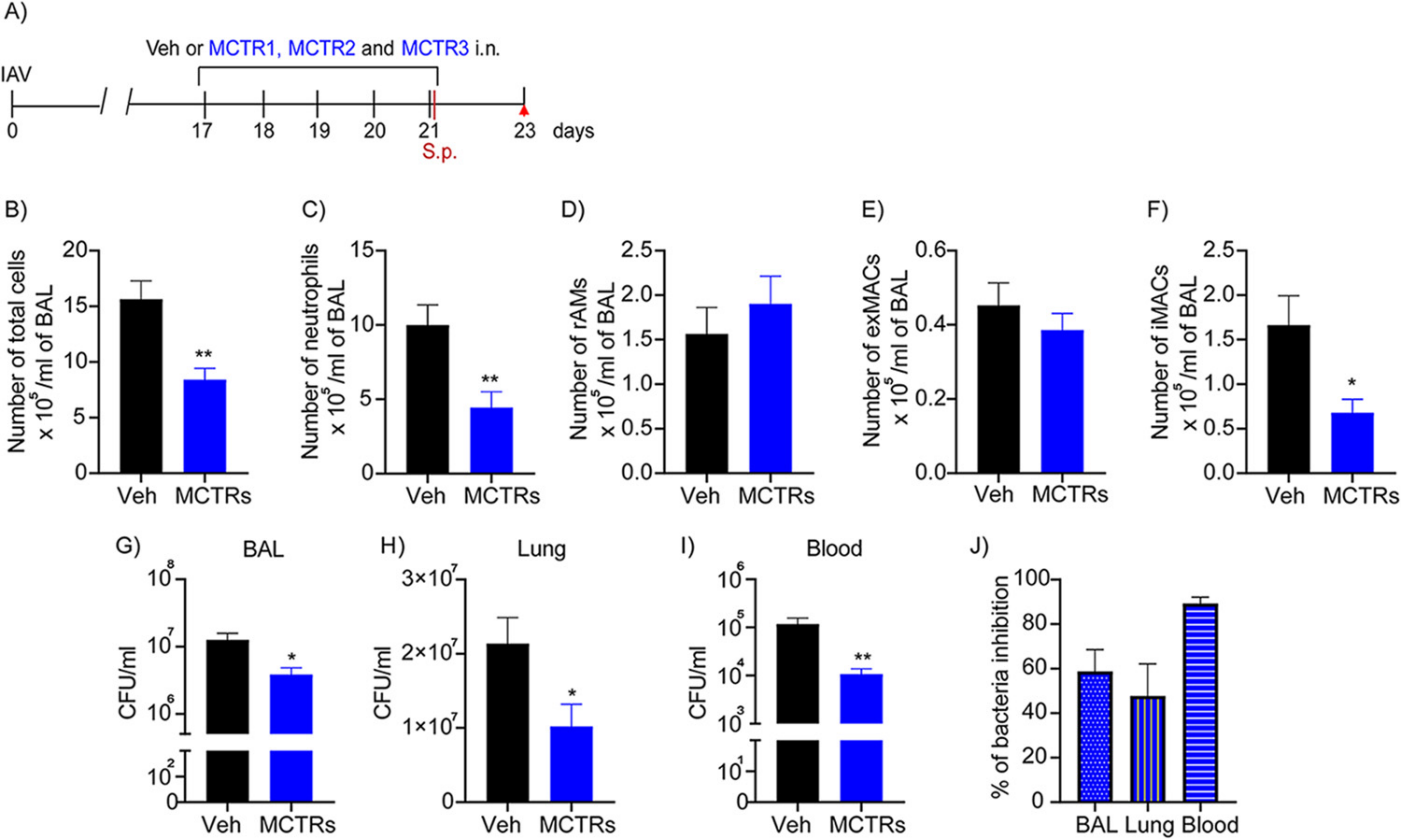
MCTRs are protective from secondary pneumococcal pneumonia post-IAV infection. (A) A mix of the three MCTRs (MCTR1, MCTR2, and MCTR3; 100 ng of each/mouse, i.n.) or vehicle was given daily on days 17 to 21 after IAV infection. On day 21 post-IAV, the animals were infected with S. pneumoniae (10^3^ CFU, i.n.) 2 h after MCTRs or vehicle. BAL was performed 48 h later, and the number of total cells (B), neutrophils (C), and macrophages (D to F) was determined by flow cytometry. (G to I) BAL fluid, left lung, and blood were used for bacterial counts. (J) The percent inhibition of bacteria CFU was calculated relative to the vehicle control. Results are expressed as mean ± SEM; *n* = 5 mice in three independent experiments. *, *P* < 0.05; **, *P* < 0.01 by unpaired Mann-Whitney test.

A mix of MCTR1 to 3 (100 ng each) was administered intranasally once daily into the airway on days 17 to 21 post-IAV infection (Fig. 4A). The day 21 administration was given 2 h prior to challenge with S. pneumoniae. Prior to the bacterial infection, the MCTR administration did not significantly alter BAL macrophage counts (rAMs, exMACs, and iMACs) (see Fig. S3B to D in the supplemental material). Of note, the MCTRs did selectively alter the macrophage phenotype. While the frequency of MCHII^high^ CD86^+^ macrophages did not change, there was a significant increase in the frequency of MCHII^low^ CD206^+^ macrophages by the MCTRs (Fig. S3I to J). After S. pneumoniae infection on day 21 post-IAV infection, the MCTRs significantly influenced the inflammatory response and bacterial burden over the subsequent 72 h (Fig. 4A; see also Fig. S3B to H). The MCTRs significantly decreased the numbers of BAL iMACs and shortened the resolution interval for 25% reduction in BAL neutrophil numbers relative to the vehicle group (Fig. 4B to F; see also Fig. S3D and E). BAL numbers of rAMs, exMACs, iMACs, and neutrophils were enumerated after S. pneumoniae infection on days 22, 23, and 24, and the MCTRs gave selective and time-dependent regulation of these cell numbers (Fig. S3B to E). In addition to the MCTR-mediated changes in lung inflammation, there were significant decreases in BAL and lung bacterial load and a marked reduction in bacteremia 48 h after lung infection (Fig. 4G to I; see also Fig. S3F to H). The percent of inhibition by MCTRs relative to vehicle was approximately 50% for BAL and lung bacterial counts and up to 90% for blood CFU (Fig. 4J). To evaluate the impact of MCTRs on host responses to primary S. pneumoniae infection, mice were infected, and 12h later, MCTRs (100 ng of each, i.n.) were administered. Relative to vehicle control, the MCTRs significantly reduced BAL rAMs, exMACs, and neutrophils 24 h after infection (see Fig. S4 in the supplemental material). Only 12 h after MCTRs, there were already trends for decreases in BAL iMAC counts and CFU. Together, these data indicate that administration of MCTRs post-IAV selectively regulated S. pneumoniae-evoked lung inflammation and enhanced bacteria host defense.

10.1128/mbio.01267-22.3 FIG S3 Kinetic analysis of immune responses and bacterial counts after secondary S. pneumoniae infection post-IAV infection. (A) A mix of all three MCTRs (MCTR1, MCTR2, and MCTR3; 100 ng of each/mouse, i.n.) or vehicle was given daily on days 17 to 21 after IAV infection. On day 21 post-IAV, a group of mice was euthanized for immunophenotyping (*n* = 6), and the other groups were infected with S. pneumoniae (10^3^ CFU, i.n.) 2 h after MCTRs or vehicle. BAL was performed 24 (*n* = 5), 48 (*n* = 5 mice in three independent experiments), and 72 h later (*n* = 4 in three independent experiments), and the numbers of macrophages (B to D) and neutrophils (E) were determined by flow cytometry. *T*_25_, interval of time to reduce 25% of the peak of neutrophil numbers; AUC, area under curve. (F to H) BAL fluid, left lung, and blood were used for bacterial counts. (I and J) The frequency of M1 (MHCII^high^ CD86^+^) and M2-like (MHCII^low^ CD206^+^) cells among rAMs (*n* = 6). Results are expressed as mean ± SEM. *, *P* < 0.05; **, *P* < 0.01; ***, *P* < 0.001 by unpaired Mann-Whitney test. Download FIG S3, TIF file, 2.3 MB. Copyright © 2022 Tavares et al. 2022 Tavares et al. https://creativecommons.org/licenses/by/4.0/ This content is distributed under the terms of the Creative Commons Attribution 4.0 International license.

10.1128/mbio.01267-22.4 FIG S4 MCTR impact on primary S. pneumoniae infection. C57BL/6 mice were infected with Streptococcus pneumoniae (10^4^ CFU) and treated 12 h later with a mix of all three MCTRs (MCTR1, MCTR2, and MCTR3; 100 ng of each/mouse, i.n.). At 24 h postinfection, BAL was performed for evaluating the numbers of macrophages (A to C) and neutrophils (D) by flow cytometry. (E) BAL was used for bacterial counts. Results are expressed as mean ± SEM. *, *P* < 0.05; **, *P* < 0.01; ***, *P* < 0.001 by unpaired *t* test. Download FIG S4, TIF file, 1.5 MB. Copyright © 2022 Tavares et al. 2022 Tavares et al. https://creativecommons.org/licenses/by/4.0/ This content is distributed under the terms of the Creative Commons Attribution 4.0 International license.

**FIG 5:**
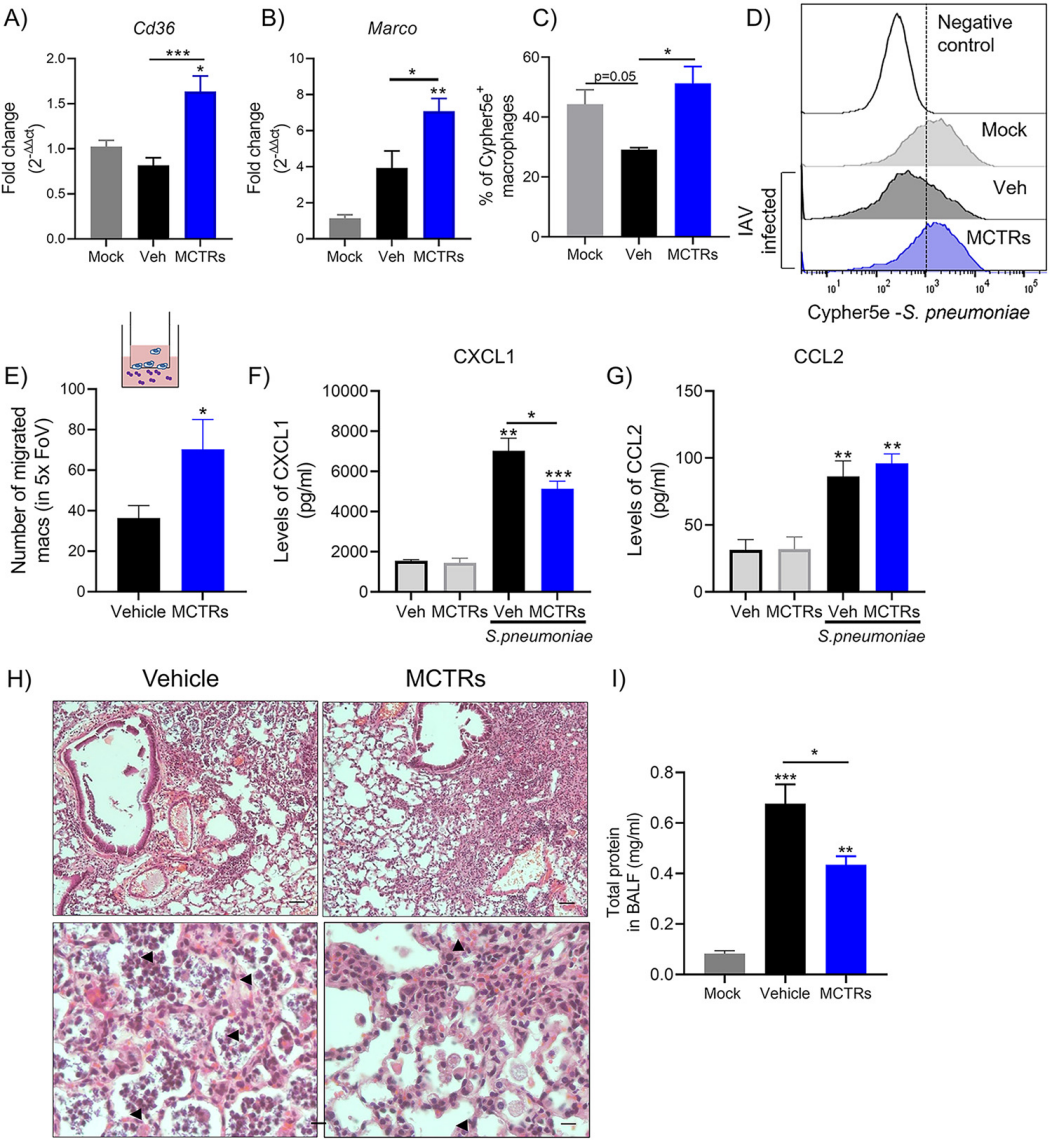
MCTR1-3 increase macrophage phagocytosis and enhance host antibacterial defenses post-IAV infection. Mice were exposed to a mix of MCTRs (100 ng each, i.n.) or vehicle (Veh) from days 17 to 21 post-IAV infection. (A and B) At day 21 post-IAV infection, quantitative PCR in the lung tissue was performed for CD36 (*Cd36*) and MARCO (*Marco*), and relative expression to uninfected (mock) was expressed as fold change (*n* = 4 in three independent experiments). (C and D) *In vivo* phagocytosis of Cypher5e-labeled S. pneumoniae (10^7^ CFU, i.n.) was evaluated after harvesting BAL cells for detection of macrophage (F4/80^+^) uptake of the labeled bacteria by flow cytometry (see Materials and Methods) (*n* = 4). (E) Cartoon of the *in vitro* migration assay and quantification of the assay of BAL macrophages from IAV-infected mice exposed to MCTRs or vehicle. S. pneumoniae was used as a stimulus for cell migration (*n* = 6). Results are presented as average of cells counted in 5 random fields of view (FoV). (F and G) A total of 10^5^ BAL macrophages was plated and stimulated *ex vivo* with S. pneumoniae (MOI, 10) for 1 h. After overnight incubation, cell supernatants were used for evaluation of CXCL1 and CCL2 levels by ELISA. (H and I) At day 21 post-IAV infection, mice were infected with S. pneumoniae and phenotyped 48 h later by lung histology (top, ×10 magnification, bars = 100 μm; bottom, ×40 magnification, bars = 50 μm) and total BALF protein levels (see Materials and Methods) (*n* = 4 in three independent experiments). Arrowheads point toward bacteria in the airways. Results are expressed as mean ± SEM. (A to C, F to I) *, *P* < 0.05; **, *P* < 0.01; ***, *P* < 0.001 by one-way ANOVA. (E) *, *P* < 0.05 by one-tailed unpaired *t* test.

Since MCTRs decreased bacterial counts in the lungs and blood, we next evaluated the expression of scavenger receptors involved in phagocytosis of S. pneumoniae (23, 25). Expression of the class B scavenger receptor CD36 and class A scavenger receptor MARCO was significantly increased in MCTR-exposed lungs post-IAV infection compared to control vehicle-exposed post-IAV infection mice as well as uninfected (mock) mice (Fig. 5A and B). To assess the functional impact of IAV and MCTRs on lung macrophages, mice were exposed to Cypher5e-labeled bacteria *in vivo* followed by quantification of bacterial ingestion by macrophages by flow cytometry. There was a trend for decreased macrophage phagocytosis of the Cypher5e-labeled S. pneumoniae at day 21 post-IAV infection compared to uninfected (mock) control macrophages (Fig. 5C and D). Administration of MCTRs *in vivo* on days 17 to 21 post-IAV infection significantly increased the frequency of Cypher5e^+^ BAL macrophages (Fig. 5C and D). *In vivo* exposure to MCTRs post-IAV infection also increased BAL macrophage transwell migration toward S. pneumoniae *in vitro* (Fig. 5E). Of interest, MCTRs selectively regulated BAL macrophage production of CXCL1, but not CCL2, after *ex vivo* stimulation with S. pneumoniae (Fig. 5F and G). Histologic analyses showed decreased neutrophilic inflammation and bacteria in the lungs from MCTR-treated mice post-IAV infection (Fig. 5H). In addition, BAL fluid (BALF) total protein was decreased after exposure to MCTRs in doubly infected mice (Fig. 5I). Together, these findings indicate that the MCTRs enhanced macrophage responses to S. pneumoniae for clearance and decreased pathogen-mediated inflammatory responses.

### MCTRs counterregulate IAV-induced transcriptional changes in alveolar macrophages.

**FIG 6:**
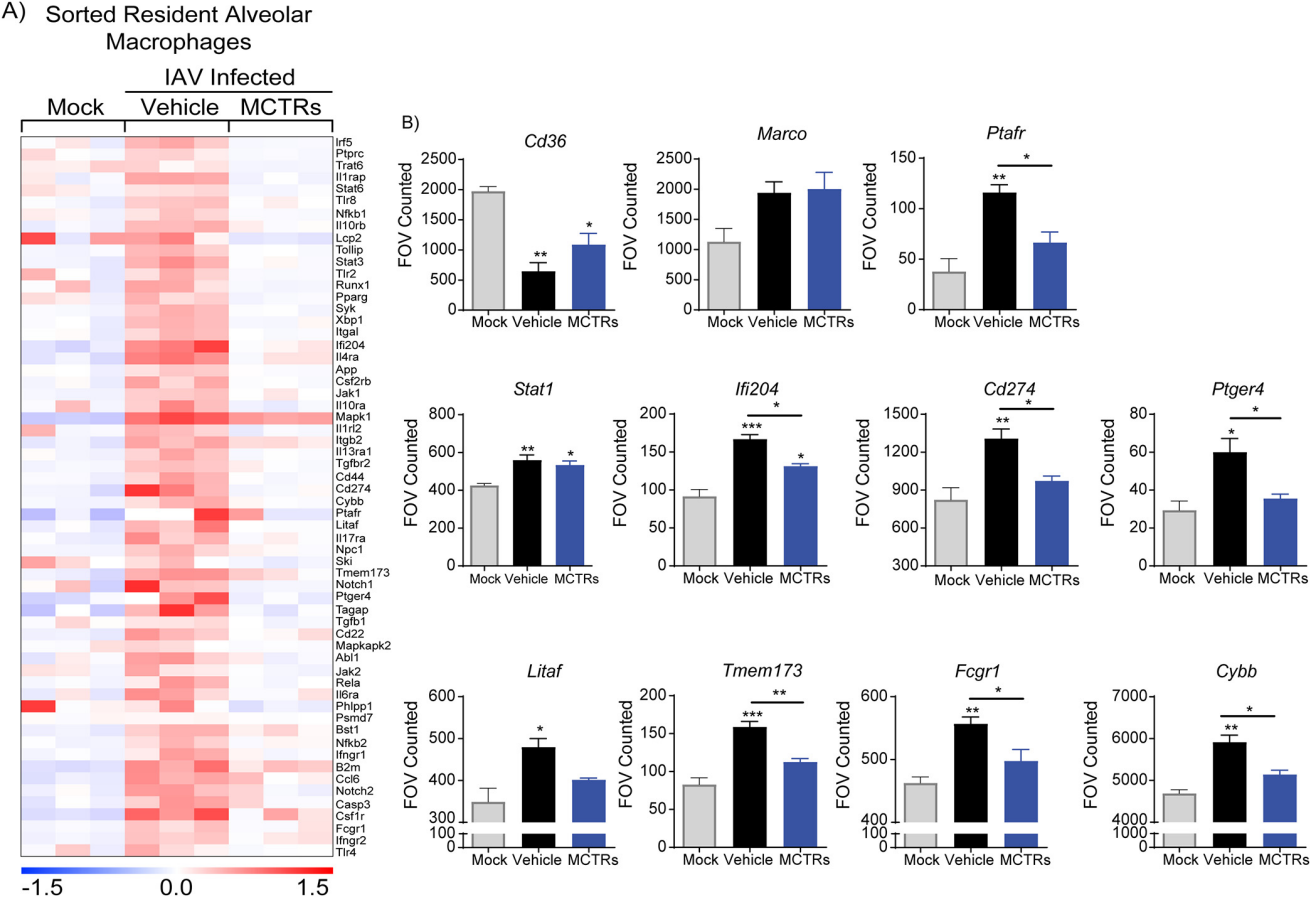
MCTRs counterregulate IAV-induced transcriptional changes in alveolar macrophage. (A) Heatmap showing relative gene expression for rAMs (F4/80^+^ CD11b^−^ CD11c^+^ SiglecF^+^) from mock (uninfected), vehicle-exposed, or MCTR-exposed mice 21 days post-IAV infection. (B) Representative genes and their relative expression after vehicle or MCTR treatment post-IAV infection. Results are presented as the normalized mRNA counts obtained. Each column in the heatmap is the result of rAMs obtained and pooled from 4 mice. Data are presented as mean ± SEM and *n* = 3 pooled samples of rAMs. *, *P* < 0.05; **, *P* < 0.01; and ***, *P* < 0.001 by one-way ANOVA.

To determine the impact of MCTRs on rAM immunophenotype 21 days post-IAV infection, inflammatory gene expression of FACS-sorted rAMs from MCTRs- or vehicle-exposed mice (days 17 to 21) was determined (Fig. 6). MCTRs markedly downregulated rAM proinflammatory gene expression (Fig. 6A). MCTRs specifically countered rAM expression of several of the pathogen-related receptors and inflammatory and interferon response genes (Fig. 6B) that were induced in rAMs 21 days post-IAV infection and related to bacterial infection susceptibility (Fig. 2). There were nonsignificant trends for increased expression of rAM *Cd36*, but not *Marco*, with MCTR exposure (Fig. 6B). Expression of *Ifi204*, *Cd274*, and *Ptger4* in rAMs were decreased by MCTRs as were genes related to inflammatory responses, such as *Ptafr*, *Litaf*, *Tmem173*, *Fcgr1*, and *Cybb* (Fig. 6B), which were upregulated post-IAV infection in comparison to those of the uninfected mock group (Fig. 2B). In contrast, other genes including *Stat1* were not affected by the MCTR treatment (Fig. 6B). Together, these data indicate that MCTRs partially reversed these IAV-altered genes in rAMs.

### MCTR3 decreases post-IAV infection secondary pneumococcal pneumonia severity.

**FIG 7:**
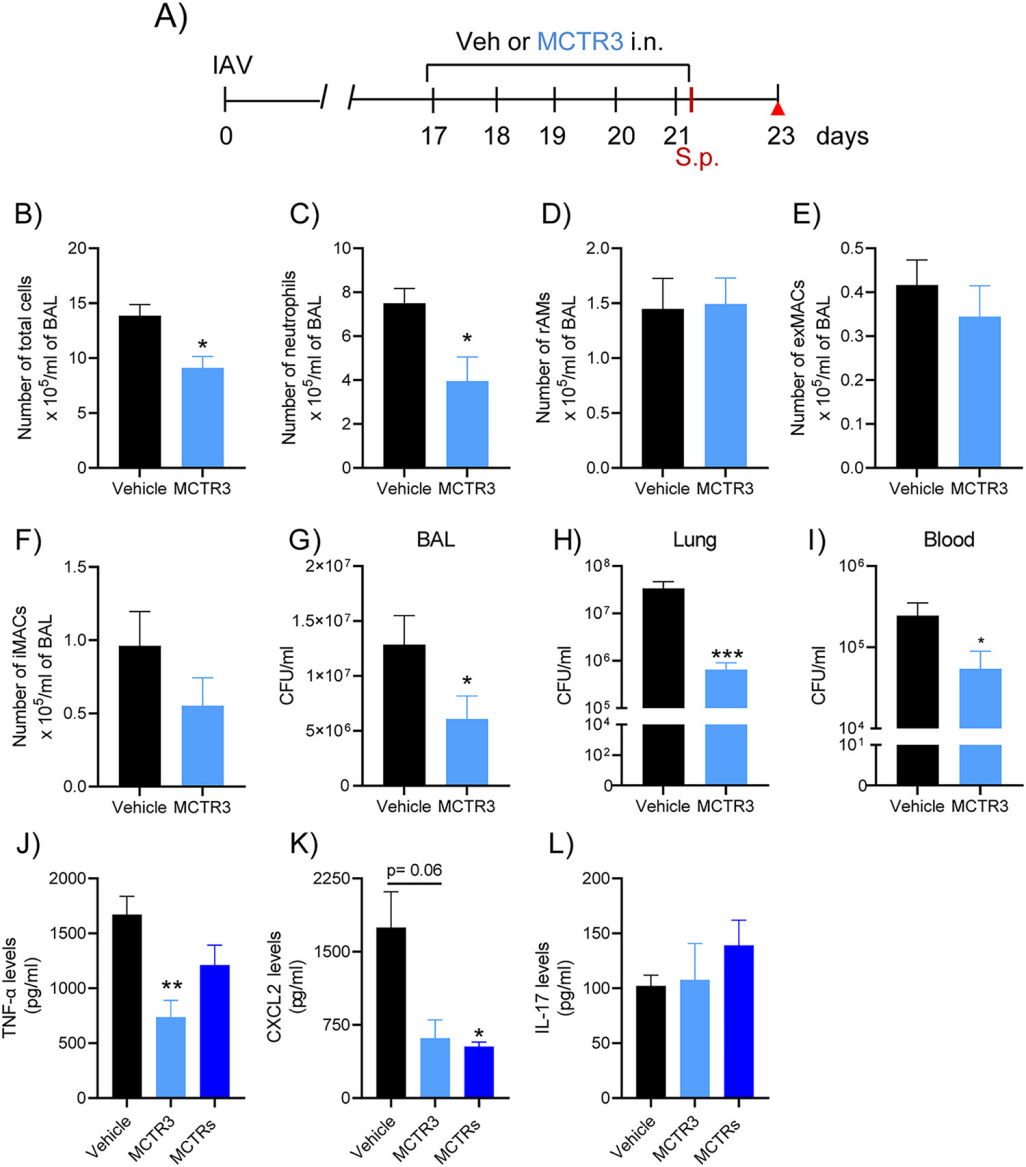
MCTR3 ameliorates pneumococcal pneumonia post-IAV infection. (A) IAV-infected mice were treated daily with MCTR3 (100 ng/mouse, i.n.) or vehicle on days 17 to 21 post-IAV infection. At day 21, the animals were infected with S. pneumoniae and 48 h later immunophenotyped. BAL total cells (B), neutrophils (C), rAMs (D), exMACs (E), and iMACs (F) were enumerated. (G to I) BAL fluid, left lung, and blood were collected for bacteria counts, and CFU were determined. *n* = 4 mice in three independent experiments; *, *P* < 0.05; and ***, *P* < 0.001 by unpaired Mann-Whitney test. (J to L) BAL levels of TNF-α, CXCL-2, and IL-17 were evaluated by ELISA (*n* = 4 to 5 mice per group). Results are expressed as mean ± SEM. *, *P* < 0.05; **, *P* < 0.01 by one-way ANOVA.

MCTR1 is converted to MCTR2, which is then converted in the lungs to MCTR3 (14). Since MCTRs can promote peritoneal macrophage phagocytosis of bacteria (13), we next investigated whether MCTR3 alone conveyed similar protective responses to those established with the combination of MCTR1, MCTR2, and MCTR3 (MCTR1 to 3). Exposure to MCTR3 (100 ng/mouse daily, i.n.) on days 17 to 21 after IAV infection (Fig. 7A) significantly decreased the number of BAL total leukocytes and BAL neutrophils 48 h after secondary pneumococcal challenge (Fig. 7B and C). BAL macrophage (rAMs, exMACs, and iMACS) numbers were similar between vehicle and MCTR3 (Fig. 7D to F). Importantly, MCTR3 treatment significantly reduced the number of bacteria in the BAL fluid, lungs, and blood of secondarily infected animals post-IAV infection (Fig. 7G and H). In addition, levels of proinflammatory cytokines were selectively regulated by MCTR3 (and similarly by the MCTRs mixture) with decreased tumor necrosis factor alpha (TNF-α) and CXCL2 levels and no significant change in interleukin-17 (IL-17) levels (Fig. 7J to L). Together, these findings indicate that MCTR3 displays similar proresolving actions for post-IAV infection secondary bacterial pneumonia to the MCTR mixture.

## DISCUSSION

Herein, we found that an IAV strain A/WSN/33 H1N1 led to long-term increased host susceptibility to pneumococcal pneumonia in C57BL/6 mice. A modest inoculum of S. pneumoniae that is well tolerated in these mice was, in sharp contrast, poorly tolerated when given 7, 14, or 21 days post-IAV infection, leading to a lethal secondary infection. Post-IAV infection, the pathogen evoked an increased inflammatory response, yet this immune response was unable to provide the requisite host defense for protection. This heightened susceptibility was associated with a transient reduction of rAMs after 7 days of infection and phenotypic and transcriptional alterations in the repopulated rAMs long after viral clearance. MCTRs are macrophage autacoid mediators that promote resolution of inflammation (13, 26). Daily administration of a mix of MCTR1 to 3 on days 17 to 21 post-IAV infection, when mice have recovered from overt manifestations of the viral infection, decreased the S. pneumoniae-elicited lung inflammation and enhanced bacterial host defense, in particular, by lung macrophages. MCTR1 and MCTR2 are enzymatically converted to MCTR3 (16) and when MCTR3 alone was administered, it displayed similar protection as all three MCTRs given together. Potential mechanisms identified here for MCTR-induced protection included reprogramming rAM gene expression to decrease proinflammatory mediators, inducing scavenger receptor gene expression, promoting macrophage migration and phagocytosis of S. pneumoniae, and modulating BAL cytokine levels.

Pneumococcal pneumonia following IAV infection evokes an intense inflammatory response in the lungs (27, 28). The sustained neutrophil recruitment together with a massive production of proinflammatory mediators leads to increased lung injury and an inability to control bacteria, culminating, in some cases, with dissemination of the pathogen and ultimately death (28, 29). In this experimental model, mice had markedly increased susceptibility to S. pneumoniae lung infection for at least 3 weeks. In agreement with our data, others have shown that IAV infection can impact the host immune system leading to sustained susceptibility to bacteria and, in humans, increased occurrence of bacterial infections at 1 to 3 weeks after peaks of IAV incidence (30, 31). Here, on days 7 and 14 post-IAV infection, the lung macrophage numbers were significantly depleted by the IAV infection and likely to be the major factor for the increased susceptibility. Of interest, by day 21, the number of lung macrophages had returned to baseline; however, they remained functionally impaired for bacterial host defense. MCTRs were given during the third week of the model to investigate their influence on the repopulated but functionally defective lung macrophages at day 21. MCTRs induced a counterregulatory gene expression program, enhanced macrophage tracking of bacteria, and increased phagocytosis of bacteria while regulating leukocyte chemokine levels and tissue accumulation. These protective actions led to a restoration of host resilience for bacterial infection.

Mouse models of IAV infection display different susceptibilities to secondary S. pneumoniae infection. These differences in host response relate to mouse and pathogen genetic strain specificity (9, 32). For instance, IAV/H1N1/WSN33 is the viral strain used here, and this strain can infect and productively replicate in macrophages (33). We established an experimental model of post-IAV pneumococcal pneumonia susceptibility using C57BL/6 mice and IAV/H1N1/WSN33 because this combination led to host rAM depletion, enabling our investigation of potential roles for SPMs and other proresolving mechanisms with the capacity for lung catabasis and restoration of host resilience for bacterial pathogens.

Given the important roles that rAMs play in S. pneumoniae host defenses, reduction in rAM numbers caused by IAV is one principal contributor to the increased susceptibility to post-IAV S. pneumoniae infection. Similar to prior reports (8), we found a significant reduction in BAL rAMs in IAV-infected mice 7 days post-IAV infection. Of interest, 14 or 21 days after IAV infection, when rAMs had returned to baseline, animals that were previously infected with IAV remained susceptible to the otherwise nonlethal S. pneumoniae lung infection. While the numbers of macrophages were restored, these repopulated lung macrophages had distinct gene expression profiles. Even 21 days post-IAV, rAMs had markedly different inflammatory gene expression and still expressed higher levels of typical proinflammatory M1 macrophage cell surface markers and lower levels of typical counterregulatory M2 cell surface markers. In humans, monocytes derived from influenza-infected patients also present an M1-like activated immunophenotype (34). Of importance, IAV infection altered several genes that have previously been related to host susceptibility to pneumococcal infections, such as *Cd36* (23), *Ptger4* (21, 35), *Ptafr* (24), *Stat1* (36), and interferon-related genes (37). CD36, a class B scavenger receptor, can mediate S. pneumoniae clearance by phagocytosis (23). IAV infection acutely reduces *CD36* expression in human monocyte-derived macrophages (38). Interestingly, we show that the expression of *Cd36* in rAMs remained significantly decreased even after 21 days post-IAV. Prostaglandin E_2_ (PGE_2_) signaling is also associated with increased susceptibility to IAV infection (35) and bacterial infection (21), inducing immunosuppression through PGE_2_ receptor 4 (EP4) receptor signaling (21, 35). Here, IAV significantly increased expression of EP4 receptor in rAMs 21 days postinfection. In addition, other lipid mediator signaling circuits can contribute to the pathological IAV host responses. For example, the platelet-activating factor receptor (Ptafr) was increased in rAMs from IAV-infected mice and is associated with increased inflammatory responses post-IAV infection (39). Together, these results provide evidence for a sustained and significant alteration in macrophage polarization post-IAV that increases susceptibility to secondary pneumococcal infections. Monocyte-derived macrophages are recruited to the lungs in response to rAM depletion caused by IAV infection and are influenced by local microenvironmental factors to express similar markers of the embryonically seeded rAMs (32, 40); however, some transcriptional and functional differences persist in a now heterogeneous mixture of lung macrophages. Among these cellular differences, the repertoire of SPM receptor expression may also differ, including receptors for MCTR1, 2, and 3, that are yet to be identified. Once the MCTR receptors are identified, further studies will be needed to clarify whether the protective actions of MCTRs post-IAV infection relate to select lung macrophage subsets of distinct developmental origin.

In some tissues, inflammatory resolution can lead to an adaptive homeostasis characterized by increased PGE_2_ production and signaling via EP2 or EP4 receptors that can suppress immune responses to subsequent challenge, including potential bacterial pathogens (21). With IAV infection, increased production of antiviral and proinflammatory cytokines, such as type I interferons (IFNs), can also result in a transient maladaptive homeostasis after viral clearance. While IFNs can efficiently restrain viral proliferation in the early stages of infection, they do so at the cost of increasing susceptibility to bacterial infection (22, 41). In addition, type I IFNs can cause a tolerogenic response in macrophages (42) that can impair macrophage capacity to control bacterial infection (22). Here, the expression of several IFN-induced genes remained significantly increased in rAMs 21 days post-IAV infection, long after viral clearance. Here, the discordant kinetics for viral clearance and pathogen-initiated inflammatory responses are indicative of an excessive and prolonged induction of genes for viral host defense that persisted long after their essential roles in viral clearance and suggest a risk window of adaptive homeostasis, where intervention with proresolving mediators that dampen IFN signaling could mitigate post-IAV risk for secondary bacterial infection. Along these lines, DHA-derived protectins, a related family of SPMs, can decrease IFN expression after mouse respiratory syncytial virus infection (43). Additionally, harnessing proresolving pathways with SPMs, including MCTR3, was recently shown to reverse leukocyte defects in patients with severe COVID-19 (44).

Resolution of pathogen-initiated inflammation is an active process with molecular and cellular effectors that enhance pathogen clearance and promote catabasis (45). Alox12 is a pivotal biosynthetic enzyme for the recently identified specialized proresolving mediator (SPM) family of MCTRs, and, of interest, levels of Alox12-derived lipid mediators inversely correlate with severity of IAV disease (15). MCTRs are present in mouse and human lungs and display counterregulatory properties for sterile lung inflammation and airway bronchoconstrictive responses to contractile agonists (14). Here, a mixture of the Alox12-derived MCTR1, MCTR2, and MCTR3 as well as MCTR3 alone were also protective for pathogen-initiated lung inflammation.

Of note, MCTR3 can also promote resolution of peritoneal Escherichia coli bacterial infection (16). Here, MCTRs reversed several transcriptional changes in pulmonary tissues post-IAV infection, including an increase in *Cd36* and *Marco* expression and a decrease in several genes associated with host susceptibility to bacterial infections. SPM mechanisms for resolution include promoting macrophage efferocytosis (20, 46) and bacterial phagocytosis (16, 20, 45). In this report, several protective mechanisms for MCTRs were identified, including extensive reprograming of gene expression in IAV-exposed macrophages to reestablish bacterial host defense, augmentation of migration toward and phagocytosis of bacteria, and decreased pathogen-elicited BAL leukocytes and cytokine levels. Although the MCTRs evoked several host protective responses, their impact on morbidity (e.g., weight loss) and mortality from bacterial pneumonia were not established here, and additional investigation will be needed once metabolically stable analogs are developed. Together, post-IAV MCTRs decreased the severity of secondary S. pneumoniae infection with significantly reduced pneumonic lung inflammation and alveolar barrier breech.

In conclusion, IAV infection with A/WSN/33 H1N1 leads to decreased rAM numbers and prolonged changes in rAM activation that contribute to susceptibility to secondary pneumococcal infection and, more generally, to delayed resolution of host inflammatory responses that persist long after viral clearance. Post-IAV infection exposure to MCTRs reprogrammed rAMs to enhance antibacterial macrophage function and improve resilience to S. pneumoniae lung infection and bacteremia. Together, these findings have further defined pivotal roles for lung macrophages in host defense and the resolution of inflammation as well as the impact of severe IAV lung infection in macrophage homeostatic functions. Pharmacological dosing of SPMs holds promise as a therapeutic strategy to augment macrophage proresolving responses to protect the host with severe respiratory viral infections from the excess morbidity and mortality of secondary bacterial infections.

## MATERIALS AND METHODS

### Mice.

C57BL/6 male mice (8 to 10 weeks old; Jackson Laboratory, Farmington, CT) were maintained with free access to commercial chow and water. Animal procedures were approved by the Animal Review Committee at Harvard Medical School (number 2016N000356) and Universidade Federal de Minas Gerais, Brazil (number 381/2015).

### Bacterial and virus strains.

Influenza A/WSN/33 H1N1 virus (IAV) was grown in Madin-Darby canine kidney (MDCK; ATCC) cells as described (39) and stored at −80°C. Titer of IAV was determined by PFU assay in MDCK monolayers as described (39).

Streptococcus pneumoniae (ATCC 6303, serotype 3) stocks were plated on blood agar plates and grown for 12 h at 37°C and 5% CO_2_. Fresh colonies were grown in 10 mL of Todd-Hewitt broth (0.5% of yeast extract) until log phase (optical density at 600 nm [OD_600_] = 0.4). After centrifugation (2,000 rpm, 20 min), bacteria were washed, resuspended in 1 mL, and diluted for infection in sterile saline. Inoculum was confirmed by plating appropriate dilutions on blood agar plates counting CFU after 12 h growth at 37°C and 5% CO_2_.

### Infection of mice.

Mice were infected intranasally with 500 PFU of IAV diluted in phosphate-buffered saline (PBS) (30 μL). After 7, 14, or 21 days of IAV infection, mice were secondarily infected with 10^3^ CFU of Streptococcus pneumoniae intranasally. Virus and bacteria singly infected mice were used as controls in addition to animals instilled with sterile PBS (uninfected, mock).

### Schedule of administration.

IAV-infected mice (day 0) were given an intranasal mixture of MCTR1 (13*R*-glutathionyl,14*S*-hydroxy-4*Z*,7*Z*,9*E*,11*E*,16*Z*,19*Z*-docosahexaenoic acid), MCTR2 (13*R*-cysteinylglycinyl,14*S*-hydroxy-4*Z*,7*Z*,9*E*,11*E*,16*Z*,19*Z*-docosahexaenoic acid), and MCTR3 (13*R*-cysteinyl,14*S*-hydroxy-4*Z*,7*Z*,9*E*,11*E*,13*R*,14*S*,16*Z*,19*Z*-docosahexaenoic acid) (100 ng/each per mouse), MCTR3 alone (100 ng per mouse), or vehicle daily, from days 17 to 21 post-IAV infection. At day 21 post-IAV infection, mice were infected with S. pneumoniae 2 h after the last treatment and euthanized 24, 48, and 72 h later (days 22, 23, and 24, respectively) to assess inflammation and bacterial counts in the lungs and blood. To evaluate the impact of MCTRs during S. pneumoniae primary infection, mice were infected with 10^4^ CFU of bacteria and after 12 h were given an intranasal mixture of MCTR1 to 3 (100 ng/each) or vehicle. At 24 h postinfection, mice were euthanized to evaluate inflammation and BAL bacterial counts.

### Tissue extraction and bronchoalveolar lavage.

At selected time points, mice were euthanized, blood was collected, and BAL performed. BAL fluid (BALF) (100 μL) was plated on blood agar for bacterial counts. After centrifugation (700 × *g*, 5 min at 4°C), the BAL cell pellet was used for total cell counts and flow cytometry analysis. BALF supernatants were used for total protein (Bradford assay) and cytokine quantification (TNF-α, CXCL-2, and IL-17) by enzyme-linked immunosorbent assay (ELISA) according to the manufacturer’s instructions. Lungs not undergoing BAL were snap-frozen in liquid nitrogen and stored at −80°C for quantitative PCR (qPCR) analyses. Right lungs were fixed with zinc fixative at a transpulmonary pressure of 20 cm H_2_O and embedded in paraffin. Five-micrometer sections of lungs were prepared and stained with hematoxylin and eosin (H&E).

### Lung RNA extraction and quantitative real-time PCR.

Right lungs were homogenized in 1 mL of TRIzol, and total RNA was extracted, as in reference 47. After DNase, cDNA was obtained with the TaqMan reverse transcription kit. Quantitative PCR was performed with the ARIA real-time PCR system (Agilent Technologies, Santa Clara, CA) using EvaGreen supermix and primers for *Cd36* and *Marco*. Quantification was carried out using Agilent MxPro and Agilent AriaMx. Results are presented as 2^−ΔΔCt^. See the supplementary material for the list of primers used (see Table S1 in the supplemental material).

10.1128/mbio.01267-22.5 TABLE S1 Primers used for qPCR analysis. Download Table S1, DOCX file, 0.01 MB. Copyright © 2022 Tavares et al. 2022 Tavares et al. https://creativecommons.org/licenses/by/4.0/ This content is distributed under the terms of the Creative Commons Attribution 4.0 International license.

### Flow cytometry and cell sorting.

BAL cells were stained with the following fluorochrome-labeled antibodies: CD45 (30-F11, PercP), F4/80 (BM8, PeCy-7), CD11c (N418, FITC), CD11b (M1/70, PE/Dazzle), Siglec F (E50-2440, PE), CD86 (GL-1, APC), CD206 (C068C2, BV605), Ly6G (1A8, Alexa-Fluor 700), and MHCII (M5/114.15.2, BV421). Neutrophils (CD45^+^ CD11b^+^ Ly6G^+^), rAMs (CD45^+^ F4/80^+^ CD11c^+^ CD11b^−^ Siglec F^+^), exudative macrophages (exMACs) (CD45^+^ F4/80 ^+^CD11c^+^ CD11b^+^), and infiltrating macrophages (iMACs) (CD45^+^ F4/80^+^ CD11c- CD11b^+^) were assessed using a BD LSR Fortessa and FlowJo v10. In separate experiments, BAL fluid from 4 to 5 animals was pooled and rAMs (F4/80^+^ CD11c^+^ CD11b^−^ Siglec F^+^) were sorted using FACS Aria. Sorted cells were used for expression data analysis (NanoString Technologies).

### Expression data analysis.

Total mRNA was isolated from sorted rAMs using the RNeasy microkit according to the manufacturer’s protocol. The NanoString panel for mouse immunology codeset (547 immune genes and 14 housekeeping genes) was used to analyze macrophage gene expression that was further quantified on the nCounter Digital Analyzer. Assay and spike-in controls were used for normalization based on identical amounts of input RNA (100 ng/sample). Counts were generated with nSolver (v4.0) software. The correlation of gene expression among cell types was calculated and plotted as a heatmap using the MultiExperiment Viewer (MeV) software and GraphPad Prism. Differentially expressed genes were identified by Student’s *t* test, and a *P* value of <0.05 was considered statistically significant. Results are presented as mRNA normalized counts obtained by the nCounter DIGITAL ANALYZER.

### Phagocytosis assay.

At day 21 post-IAV infection, log-phase S. pneumoniae was labeled with the pH-sensitive dye Cypher5e (GE Healthcare Life Sciences) for 30 min at room temperature. Approximately 10^7^ CFU of labeled bacteria were administered intranasally. The inoculum was confirmed by growth on blood agar plates. Four hours later, BAL was performed, cells were stained with F4/80 (PeCy-7), and flow cytometry was performed. Results are presented as the frequency of Cypher5e^+^ macrophages (F4/80^+^) among total cells.

### Structural validation of MCTR1, MCTR2, and MCTR3.

MCTRs were prepared by total organic synthesis (13) by N. A. Petasis (Department of Chemistry, University of Southern California) via subcontract for P01GM095467 (Charles N. Serhan). MCTR1 to 3 were each validated and confirmed by identical retention times and unbiased library fits to reference materials using a custom cys-SPM spectral MS/MS library. Data were acquired on an ExionLC coupled to a 6500+ triple quadrupole QTRAP mass spectrometer operated in low-mass positive mode using Analyst 1.7.1. The library was constructed using LibraryView version 1.4, and synthetic materials were validated against authentic materials. Library matching parameters are listed in the supplemental material. The structural features of the compounds are consistent with those originally reported for MCTR1 to 3 (16) (Text S1).

10.1128/mbio.01267-22.6 TEXT S1 Methodology for the structural validation of MCTR1, MCTR2, and MCTR3. Download Text S1, DOCX file, 0.01 MB. Copyright © 2022 Tavares et al. 2022 Tavares et al. https://creativecommons.org/licenses/by/4.0/ This content is distributed under the terms of the Creative Commons Attribution 4.0 International license.

### *In vitro* migration assay.

Harvested BAL macrophages were counted, and a migration assay was performed using transwell inserts with 8-μm pores (VWR). Briefly, 3 × 10^5^ macrophages were added to the top chamber, and 5 × 10^6^ CFU of S. pneumoniae in RPMI 1640 supplemented with 5% fresh mouse serum was added to the bottom chamber. Cells were allowed to migrate for 6 h (37°C, 5% CO_2_). Transwell inserts were fixed, permeabilized with methanol, and stained with methylene blue. Cells on the input side of the membrane were scraped off, and membranes were mounted on microscopy slides. Results are presented as the average of cell counts in 5 random high power (100×) fields of view.

### *Ex vivo* stimulation of macrophages.

BAL macrophages from IAV-infected mice exposed to MCTRs or vehicle were harvested on day 21 postinfection. A total of 100,000 macrophages were isolated by adhesion in 96-well plates, washed with Dulbecco modified Eagle medium (DMEM), and stimulated with live S. pneumoniae (multiplicity of infection [MOI], 1:10) for 1 h. Next, the cells were washed and incubated overnight at 37°C and 5% of CO_2_. Cell culture supernatants were harvested and used for the analysis of CXCL1 and CCL2 levels by ELISA per the manufacturer’s instructions.

### Statistics.

Results are expressed as mean ± standard error of the mean (SEM). Differences between two groups were calculated by two-tailed unpaired Student’s *t* test or Mann-Whitney U test when data distribution was nonnormal. One-way analysis of variance (ANOVA), followed by a *post hoc* test, was used to compare more than two groups. A *P* of <0.05 was considered statistically significant. Statistics were performed using GraphPad Prism version 8.0.0 for Windows (GraphPad Software, San Diego, CA, USA).
