# Supplementary figures and images for: Cysteinyl Maresins Reprogram Macrophages to Protect Mice from Streptococcus pneumoniae after Influenza A Virus Infection
Source: mBio. 2022 Aug 1;13(4):e01267-22. doi: 10.1128/mbio.01267-22 (PMC9426576; doi:10.1128/mbio.01267-22)

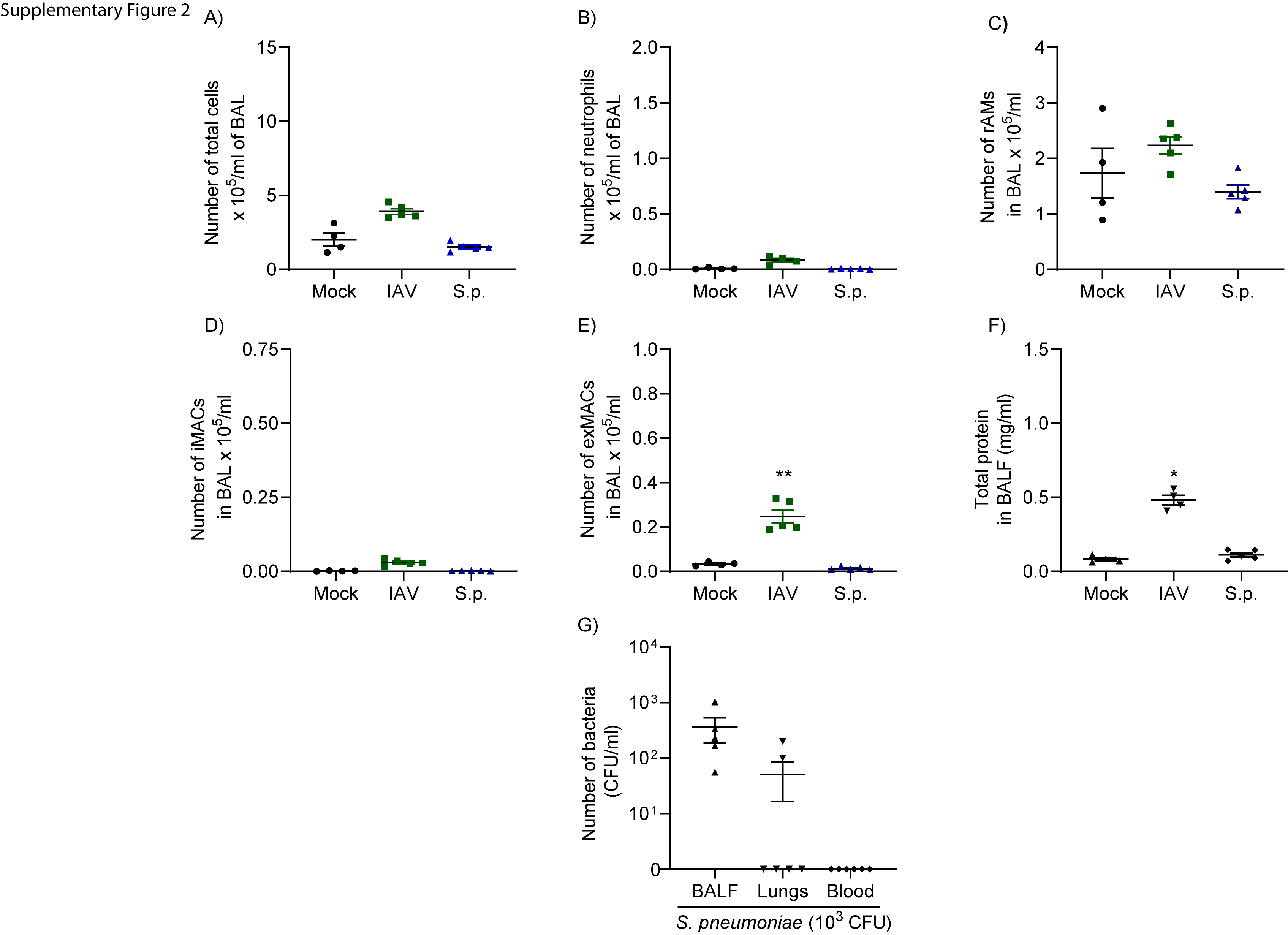

Supplement: FIG S2 [file mbio.01267-22-s0002.tif]

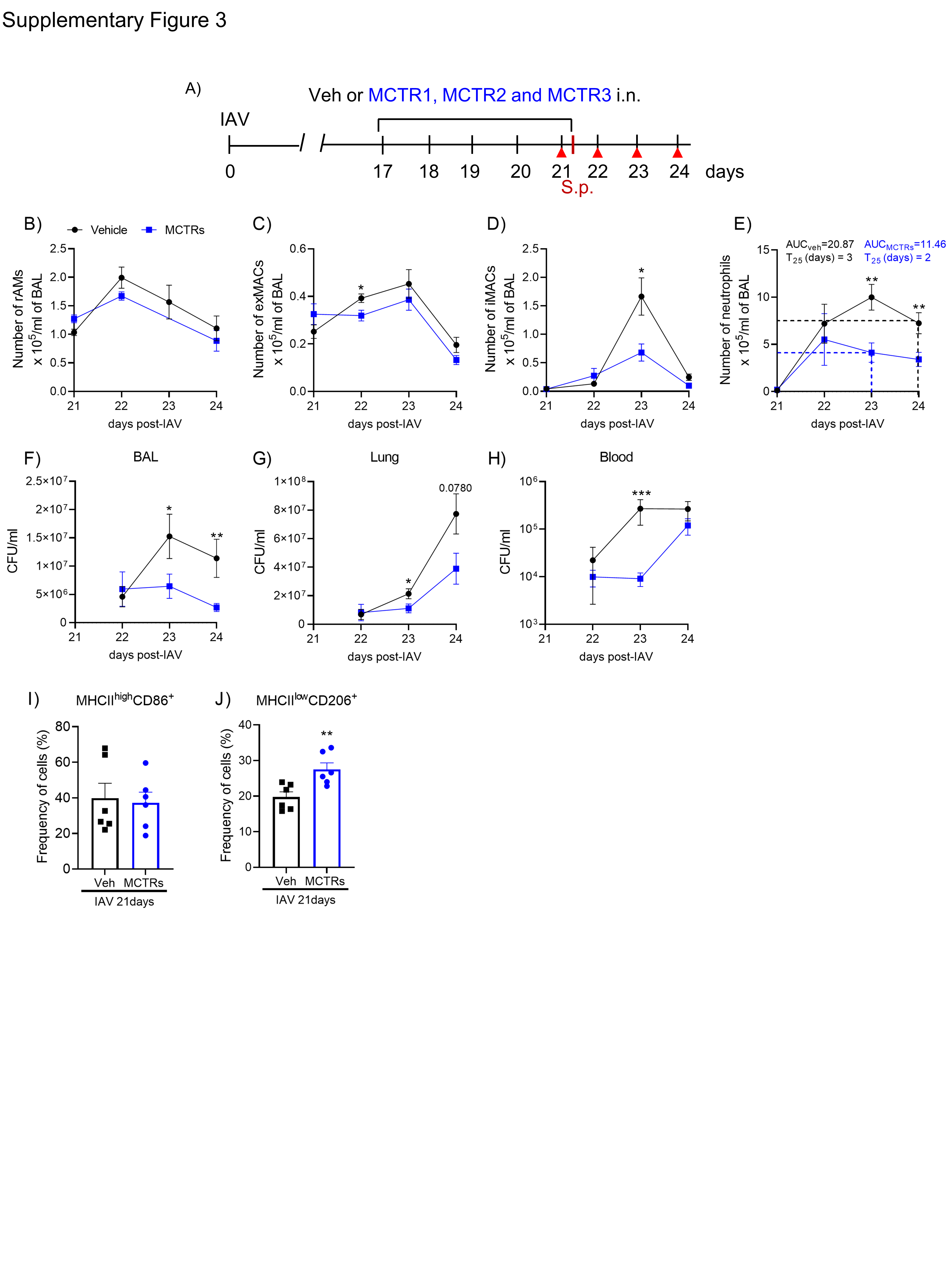

Supplement: FIG S3 [file mbio.01267-22-s0003.tif]

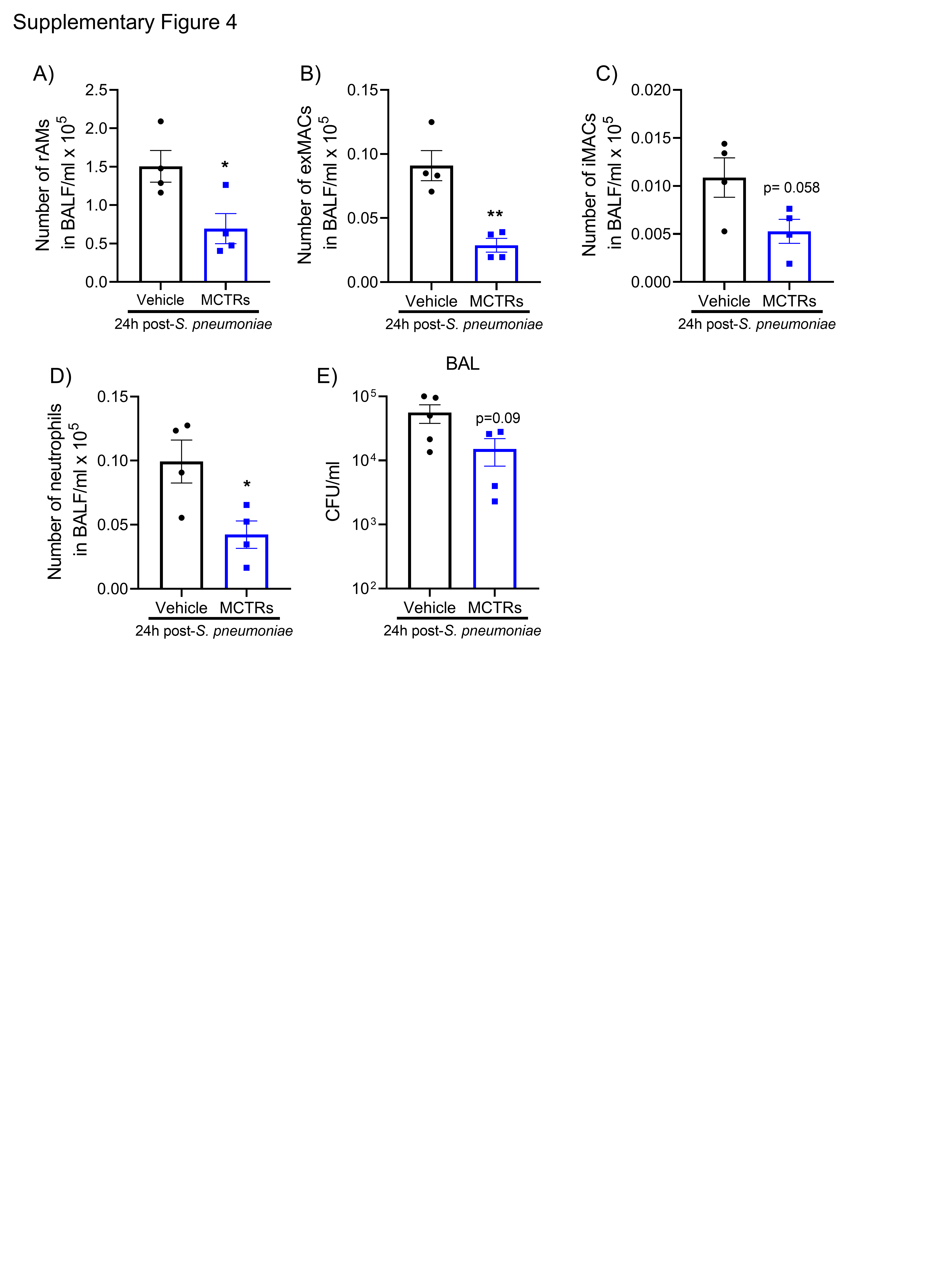

Supplement: FIG S4 [file mbio.01267-22-s0004.tif]
