# Supplementary material for: Cysteinyl Maresins Reprogram Macrophages to Protect Mice from Streptococcus pneumoniae after Influenza A Virus Infection
Source: mBio. 2022 Aug 1;13(4):e01267-22. doi: 10.1128/mbio.01267-22 (PMC9426576; doi:10.1128/mbio.01267-22)
Supplement: TABLE S1 [file mbio.01267-22-s0005.docx]

**Supplementary Table 1: Primers used for qPCR analysis.**

| **Gene** | **Primer Sequences** |
| --- | --- |
| ***Cd36*** | **Fw:** 5’- CCT CCA GAA TCC AGA CAA CC-3’  **Rv:** 5’- CAC AGG CTT TCC TTC TTT GC-3’ |
| ***Gapdh*** | **Fw:** 5’- CGT CCC GTA GAC AAA ATG G-3’  **Rv:** 5’- TTG ATG GCA ACA ATC TCC AC-3’ |
| ***M1*** | **Fw:** 5’- TGC AAA AAC ATC TTC AAG TCT CTG -3’  **Rv:** 5’- AGA TGA GTC TTC TAA CCG AGG TCG -3’ |
| ***Marco*** | **Fw:** 5’- TCC CTG TGA TGG AGA CCT TC-3’  **Rv:** 5’- GTG AGC AGG ATC AGG TGG AT-3’ |
