# Supplementary material for: Cysteinyl Maresins Reprogram Macrophages to Protect Mice from Streptococcus pneumoniae after Influenza A Virus Infection
Source: mBio. 2022 Aug 1;13(4):e01267-22. doi: 10.1128/mbio.01267-22 (PMC9426576; doi:10.1128/mbio.01267-22)
Supplement: TEXT S1 [file mbio.01267-22-s0006.docx]

SUPPLEMENTARY METHODS:

**Structural Validation of MCTR1, MCTR2, and MCTR3:**

Library matching parameters were as follows: precursor mass tolerance = ± 0.8 Da, collision energy = ± 5 eV, fragment mass tolerance = ± 0.4 Da, use polarity, intensity threshold = 0.02, minimal purity = 5.0 %, and intensity factor = 100. For MCTR1: Q1 = 650.3 Da, Q3 = 191.1 Da, dwell time = 200 msec, declustering potential = 40 V, entrance potential = 10 V, collision energy = 28 V, and collision cell exit potential = 13 V. For MCTR2: Q1 = 521.3 Da, Q3 = 191.1 Da, dwell time = 200 msec, declustering potential = 40 V, entrance potential = 10 V, collision energy = 23 V, and collision cell exit potential = 13 V. For MCTR3: Q1 = 464.3 Da, Q3 = 191.1 Da, dwell time = 200 msec, declustering potential = 40 V, entrance potential = 10 V, collision energy = 25 V, and collision cell exit potential = 13 V. For multiple reaction monitoring (MRM), enhanced product ion (EPI), column, and gradient settings (45). Screen captures of targeted MRM and EPI scans were obtained from Sciex OS-Q version 1.7.0.36606.
